# Adaptive clinical trials in surgery: A scoping review of methodological and reporting quality

**DOI:** 10.1371/journal.pone.0299494

**Published:** 2024-05-28

**Authors:** Phillip Staibano, Emily Oulousian, Tyler McKechnie, Alex Thabane, Samuel Luo, Michael K. Gupta, Han Zhang, Jesse D. Pasternak, Michael Au, Sameer Parpia, J. E. M. (Ted) Young, Mohit Bhandari

**Affiliations:** 1 Division of Otolaryngology–Head and Neck Surgery, Department of Surgery, McMaster University, Hamilton, Ontario, Canada; 2 Department of Health Research Methodology, Evidence, and Impact, McMaster University, Hamilton, Ontario, Canada; 3 McGill University School of Medicine, McGill University, Montreal, Quebec, Canada; 4 Division of General Surgery, Department of Surgery, McMaster University, Hamilton, Ontario, Canada; 5 Michael G. DeGroote School of Medicine, Hamilton, Ontario, Canada; 6 Endocrine Surgery Section Head, Division of General Surgery, Department of Surgery, University Health Network, University of Toronto, Toronto, Ontario, Canada; 7 Division of Orthopedic Surgery, Department of Surgery, McMaster University, Hamilton, Ontario, Canada; University of Toronto, CANADA

## Abstract

**Importance:**

Adaptive surgical trials are scarce, but adopting these methods may help elevate the quality of surgical research when large-scale RCTs are impractical.

**Objective:**

Randomized-controlled trials (RCTs) are the gold standard for evidence-based healthcare. Despite an increase in the number of RCTs, the number of surgical trials remains unchanged. Adaptive clinical trials can streamline trial design and time to trial reporting. The advantages identified for ACTs may help to improve the quality of future surgical trials. We present a scoping review of the methodological and reporting quality of adaptive surgical trials.

**Evidence review:**

We performed a search of Ovid, Web of Science, and Cochrane Collaboration for all adaptive surgical RCTs performed from database inception to October 12, 2023. We included any published trials that had at least one surgical arm. All review and abstraction were performed in duplicate. Risk of bias (RoB) was assessed using the RoB 2.0 instrument and reporting quality was evaluated using CONSORT ACE 2020. All results were analyzed using descriptive methods.

**Findings:**

Of the 1338 studies identified, six trials met inclusion criteria. Trials were performed in cardiothoracic, oral, orthopedic, and urological surgery. The most common type of adaptive trial was group sequential design with pre-specified interim analyses planned for efficacy, futility, and/or sample size re-estimation. Two trials did use statistical simulations. Our risk of bias evaluation identified a high risk of bias in 50% of included trials. Reporting quality was heterogeneous regarding trial design and outcome assessment and details in relation to randomization and blinding concealment.

**Conclusion and relevance:**

Surgical trialists should consider implementing adaptive components to help improve patient recruitment and reduce trial duration. Reporting of future adaptive trials must adhere to existing CONSORT ACE 2020 guidelines. Future research is needed to optimize standardization of adaptive methods across medicine and surgery.

## Introduction

Randomized-controlled trials (RCTs) are essential for evaluating the effectiveness and safety of interventions in healthcare [1]. Their importance is reflected in the literature: since 1965, over 39,000 RCTs have been published globally, with over 60% published in the last 20 years [2]. In 2003, however, only 3.4% of studies published in leading journals were surgical RCTs [3]. Despite a 50% increase in the number of published surgical trials between 1999 and 2009, this number has remained stable over the past decade [4]. Surgical trials suffer from a high rate of discontinuation and nonpublication rates often due to slow patient recruitment [5]. A systematic review of surgical trials published from 2008 to 2020 highlighted several methodological concerns with surgical RCTs, including small sample sizes, a focus on minor clinical outcomes, moderate-to-high bias, and inconsistent usage of blinding and expertise-based randomization [3, 6].

In medicine, at least 50% of adopted interventions are derived from RCTs, yet fewer than 25% of surgical interventions are based on evidence derived from RCTs [3]. Adherence to clinical trial methodological standards in surgery is often impacted by high costs, feasibility issues, between-group crossover, and poor patient adherence [7, 8]. As a consequence, many surgical innovations have been adopted based upon non-scientific practices and small-scale, poorly controlled observational studies [6]. Randomized studies in surgery, however, have historically led to an effective discarding of unnecessary surgical procedures [9].

The conventional randomized trial design with a large sample size remains the gold-standard approach for comparing medical interventions. Classically, RCTs adhere to a fixed study protocol and culminate in a pre-defined final analysis. Adaptive clinical trials, however, involve flexible adjustments to the study protocol based upon pre-specified interim analyses, which can permit sample size re-calculations, adding or dropping treatment arms, and/or stopping the trial for futility or lack of efficacy (Table 1). Adaptive trials are gaining popularity in drug development and other medical disciplines, as demonstrated by the TAILoR, 18-F PET, and STAMPEDE trials [10–12]. Benefits of trial adaptability include cost reduction, decreased probability of assigning patients to an ineffective treatment arm, and expedited trial completion [13]. Adaptive trials were useful during the peak of COVID-19 to accelerate the comparison of anti-viral therapies [14]. Adaptive methodology, however, has been less adopted in evidence-based surgery. Given the challenges surgical researchers face in implementing conventional RCTs, adaptive trials may represent a high-quality alternative that allows surgical researchers to retain the benefits of randomization whilst minimizing costs and permitting protocol adjustments to maximize trial feasibility, adherence, and validity. The goal of this study was to perform a scoping review to characterize the methodological and reporting quality of adaptive study designs in surgical trials. These findings will define the current landscape of adaptive surgical trial quality so that they can be optimized and applied to future surgical populations.

**Table 1:** Advantages and disadvantages of adaptive trial designs.

Advantages	Disadvantages
Early study termination or dropping treatment arm due to efficacy, futility, or harm. Improve statistical power. Maximize probability that patient is recruited to better-performing treatment group. Reduce costs associated with trial design and implementation. Reduce the sample size. Reduce the number of trials needed to address a clinical question. Reduce time to trial reporting.	Difficult to secure funding. Less applicable if delayed time to outcome assessment. Risk of introducing bias if not well-designed Statistically onerous Requires reliable research infrastructure.

## Materials and methods

### Study design

We performed a scoping review of all prospective randomized trials that have employed adaptive designs within any surgical discipline. Surgical disciplines for the purposes of this study included cardiothoracic surgery, general surgery, gynecological surgery, orthopedic surgery, ophthalmology, otolaryngology, neurosurgery, plastic surgery, and urological surgery. We included all studies that compared at least one surgical arm. We also included studies that compared at least one surgical arm to a non-surgical interventional procedure. We registered this review in Open Science Framework (DOI: 10.17605/OSF.IO/2GDC5). Due to the nature of this study, institutional review board (IRB) approval was not required. This review was performed in accordance with PRISMA Scoping Review guidelines [15].

### Search strategy

We performed a library citation search of Medline (Ovid), EMBASE (Ovid), Web of Science, Cochrane Collaboration, and CENTRAL databases from inception to October 14th, 2023 (S1 File). The search strategy was finalized by a librarian specialist. We performed a search of pre-print databases to identify any relevant drafted manuscripts or ongoing clinical trials. We also performed a search of clinicaltrials.gov using the keywords “adaptive/Bayesian”, “clinical trial/trial”, and “surgery/surgical” to evaluate for any active surgical trials meeting our eligibility criteria. We also used keywords for each included surgical discipline. We screened the first 10 pages of relevant results of clinicaltrials.gov and pre-publication databases. Additional studies were identified through reference list searches of included articles. All duplicates were removed, and citations were managed using Covidence software (Melbourne, Victoria, Australia) [16].

### Study selection

Any adaptive trial investigating one or more surgical or procedural interventions was included. Eligible trials must have included one of the following adaptive designs: Bayesian, frequentist, sample re-estimation, group sequential, multi-arm multi-stage (MAMS), seamless, continual reassessment, population enrichment, adaptive randomization, and/or adaptive dose-ranging. Any protocols for adaptive surgical trials were collated for the discussion, but not included in the final article synthesis. We excluded any trials that did not include at least one surgical arm, as well as any trials evaluating perioperative medications or non-surgical interventions provided in a surgical setting. Abstracts and conference proceedings, non-human studies, and non-English publications were also excluded.

### Outcomes of interest and data abstraction

The primary outcome of this study was to characterize the existing adaptive surgical trial literature and assess the methodological and reporting quality. All identified citations underwent screening of titles and abstracts in duplicate (E.O. and S.L.), followed by full-text evaluation in duplicate (P.S. and E.O.). With the use of a standardised and piloted data abstraction template, the following study characteristics were extracted: publication year, country of study, study design, and methodological details pertaining to adaptive design. We used CONSORT ACE 2020 to guide data abstraction and identified the following adaptive trial characteristics: type of adaptive design, number and type of pre-determined interim analyses, goals of interim analysis, presence of any statistical simulations, and details related to randomization, blinding, type I error adjustments, and final statistical analysis [17]. All data abstraction was performed in duplicate (E.O. and S.L.) and any conflicts resolved by third reviewer (P.S.).

### Quality appraisal

For all studies meeting eligibility criteria, we performed quality appraisal using the Cochrane Risk-of-Bias for Randomized Trials (RoB 2.0) instrument [18]. We also performed a reporting quality appraisal using the CONSORT ACE 2020 guidelines for adaptive trials [17]. The author checklist was applied to the abstract and main text for all included articles. We categorized quality of reporting into *fully reported*, *partially reported (with details provided*, *where relevant)*, and *not reported*. All quality appraisal was performed in duplicate (E.O. and S.L.). All conflicts were resolved via discussion and a third reviewer (P.S.).

### Statistical analysis

We performed descriptive statistical analysis for all included studies. We reported all continuous outcomes as means (with standard deviation) or median (with ranges), where applicable. All categorical outcomes were reported as proportions and percentages, where applicable. All analyses were performed in Microsoft Excel (Redmond, Washington, USA).

## Results

### Search strategy and article selection

**Fig 1:**
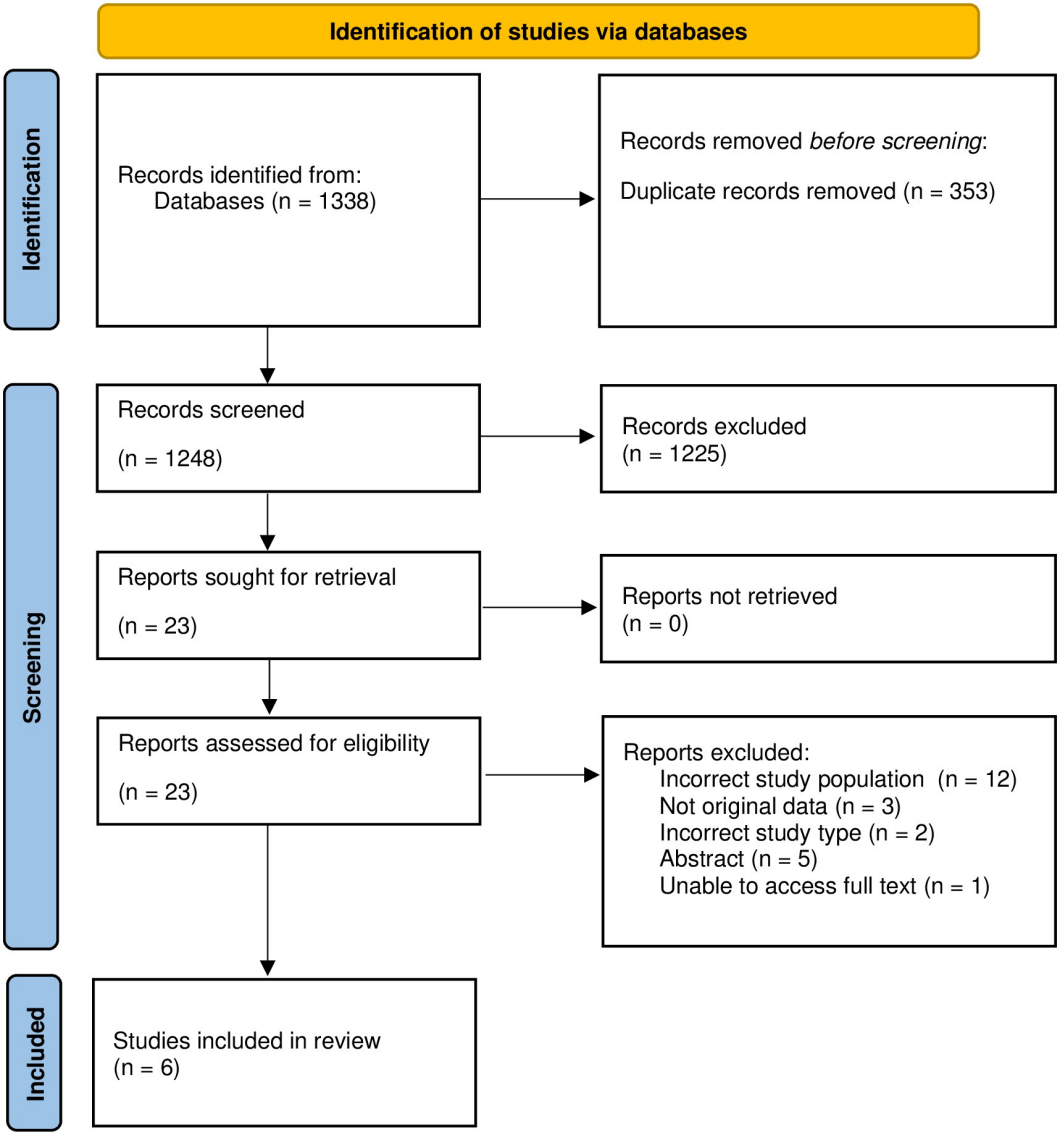
PRISMA flow chart for study inclusion.

Our database search yielded 1338 results from database inception to October 2023, of which, six published trials met eligibility criteria (i.e., <0.5% of retrieved citations) (Fig 1) [19–24]. Our review of clinicaltrials.gov yielded 263 active trials, but none met the eligibility criteria. Our review of pre-publication databases did not yield any manuscripts that met eligibility criteria.

### Study characteristics

All six included studies were published after 2015 (Table 2) [19–24]. They were in the fields of cardiothoracic surgery, orthopedics, urology, and oral and maxillofacial surgery. Five studies published their study protocol in advance of trial registration [19–23]. All studies compared two treatment arms. Two trials were conducted in the US (33.3%), one in Japan (16.7%), and three in Europe (50%). Two trials were multi-institutional [21, 23]. The total number of recruited patients was 2980 and the range of patient recruitment was 30–1746 patients. We also note that three trials recruited under 150 patients [20–22]. All studies utilized at least one surgical treatment arm. One cardiothoracic surgery trial utilized an interventional procedure as an experimental arm [23]. All studies utilized 1:1 randomization, and blinding included a combination of patients, outcome assessors, and/or surgeons. These studies did not report an expertise-based group allocation design. Primary outcomes in five trials clinical endpoints of direct patient relevance [19–23]. In the five studies that reported secondary outcomes, the majority were further clinical endpoints with two studies evaluating patient-centred outcomes, and one performing a global economic analysis. The study follow-up range for all trials was 1–24 months with most studies using a 6–12-month study follow-up period. All study data is included in S2 File.

**Table 2:** Summary of included studies.

Author (year)	Study design	Country	Surgical field	No. of centres	No. patients recruited	No. patients analysed	Treatment groups		Primary outcome	Secondary outcomes	Type of adaptive design	Blinding	Data analysis plan	Type of randomization	Study follow-up	Trial adjustments made at interim analysis	Published protocol
							Experimental	Control									
Gaudino et al. (2021) [19]	Prospective RCT (superiority)	USA	Cardiothoracic surgery	1	420	420	Posterior left pericardiotomy (n = 212)	No intervention (n = 208)	Rate of postoperative atrial fibrillation during hospital admission	Cumulative time spent in atrial fibrillation, need for antiarrhythmic medications and systemic anticoagulation, the need for cardioversion, hospital readmission, and the duration of hospital admission	Group sequential; sample size re-estimation; two pre-specified efficacy interim analyses	Patients and outcome assessors	ITT; sensitivity analysis as per as-treated principle	1:1…	1 month	Sample size increased at second interim analysis due to lower-than-expected rate of primary outcome	Yes
Mastroianni et al. (2022) [20]	Prospective RCT (superiority)	Italy	Urology	1	116	116	Robot-assisted radical cystectomy (n = 58)	Open radical cystectomy (n = 58)	Perioperative transfusion rate reduction	Perioperative outcomes during hospital stay, complication rates, global cost analysis, and 6-month functional, oncologic and HRQoL outcomes	Covariate adaptive randomization	Outcome assessors (i.e., pathologists who assessed surgical specimens)	None reported		6 months	None reported	Yes
Metcalfe et al. (2022) [21]	Prospective RCT (superiority)	UK	Orthopedic surgery	24	117	114	Debridement with device (In-Space© balloon) (n = 56)	Debridement only (n = 61)	Oxford Shoulder Score at 12 months postoperatively	Constant Score, range of pain-free flexion/abduction, Western Ontario Rotator Cuff index, EuroQol EQ-5D-5L, change in symptoms, participant global impression of change, resource use, and adverse events	Group sequential; sample size re-estimation; two pre-specified interim analyses for futility and efficacy and futility, respectively	Patients (until 12 months after surgery and outcome assessors	mITT		12 months	Recruitment and randomisation stopped at the first interim analysis based on futility criteria	Yes
Neuberger et al. (2023) [22]	Prospective RCT (superiority)	Germany	Urology	1	551	544	TP-RARP with peritoneal flap (n = 270)	TP-RARP without peritoneal flap (n = 274)	Rate of symptomatic lymphoceles needing surgical intervention	Asymptomatic lymphoceles, perioperative parameters (i.e. length of hospital stay), and postoperative complications	Group sequential; sample size re-estimation; one pre-specified interim analysis for futility and efficacy	Surgeons blinded intraoperatively until end of surgery, patients and outcome assessors were blinded for the entirety of the trial	ITT; per-protocol and as-treated for primary outcome		6 months	Sample size increased at interim analysis	Yes
Reardon et al. (2017) [23]	Prospective RCT (non-inferiority)	Canada, Europe, USA	Cardiothoracic surgery	87	1746	1,660	TAVR (n = 879)	Surgical AVR (n = 867)	Composite of death from any cause or disabling stroke at 24 months	Major adverse cardiovascular and cerebrovascular events, myocardial infarction, all types of strokes, any surgical reintervention	Bayesian design and analysis	Not reported	mITT; sensitivity analysis performed for patients lost to follow up		24 months	None reported	Yes
Yoshioka et al. (2018) [24]	Prospective RCT (superiority)	Japan	Oral and Maxillofacial Surgery	1	30	30	Intraoral vertical ramus osteotomy (n = 15)	Sagittal split ramus osteotomy (n = 15)	Cephalogram measurements of proximal and distal osteotomy segments	None reported	Adaptive randomization	NR	None reported	NR	12 months	None reported	No

HRQoL, health-related quality of life; ITT, intention-to-treat; mITT, modified intention-to-treat; NR, not reported; RCT, randomized clinical trial; TAVR, trancatheter aortic valve replacement; TP-RARP, Transperitoneal robotic assisted radical prostatectomy

### Adaptive methodology characteristics

All included studies were prospective adaptive RCTs (Table 3). One study specified a non-inferiority design [23]. Two studies employed adaptive randomization [20, 24]. Mastroianni et al. (2021) utilized covariate adaptive randomization and identified their pre-specified prognostic strata in their methods [20]. Yoshioka et al. (2018) did not describe their adaptive randomization methods [24]. Metcalfe et al. (2022) and Reardon et al. (2017) mentioned the role of statistical simulations in informing their trial design and analysis plan [21, 23]. Four studies described their proposed statistical analysis plan, including the type of group allocation analysis and the relevant inferential testing methods. Reardon et al. (2017) was the only study to employ a Bayesian method for trial design and analysis, and this group also used a statistical consulting firm to assist with calculations [23]. Three studies described a group-sequential design with two treatment arms alongside sample size re-estimation performed at each interim analysis. Four studies also described their adaptive methodologies, including the number and goal(s) of their interim analyses [19, 21–23]. These interim analyses were all pre-specified and performed for the purpose of study termination for futility or efficacy, and/or sample size re-estimation. In two studies, the sample size was increased at the interim analysis [19, 22]. Two studies utilized two pre-specified interim analyses [19, 21]. Gaudino et al. (2021) reported a sample size increase at the second interim analysis due to a lower-than-expected primary outcome event rate [19]. Metcalfe et al. (2022) stopped further randomization due to futility criteria being met [21].

**Table 3:** Adaptive design characteristics for included studies.

Author (year)	Adaptive design	Prespecified interim analysis	Goals of interim analysis	Change(s) to design after start of trial	Statistical simulations	Goals of simulation	Modelling algorithm	Sample size calculation	Type of randomization	Blinding	Type of statistical analysis	Type I error adjustments	Adaptive design reporting	Statistical resources
Gaudino et al. (2021) [19]	Group sequential; sample size re-estimation	Sample-size re-estimation Two pre-specified blinded efficacy interim analyses No futility interim analysis	Sample size re-estimation	Second interim analysis: Primary outcome event rate lower than anticipated and so, trial sample size was increased	None reported	NR	Not reported	Yes, 90% power to detect 50% reduction in the primary outcome; based on previous studies	1:1; mixed-block randomisation sequence	1. Patients and outcome assessors 2. All parties unblinded after study completion 3. Premature unmasking if complications secondary to treatment group	1. ITT for primary outcome 2. Sensitivity analysis for primary outcome per as-treated principle 3. ITT for secondary outcomes 4. As-treated principle for safety outcomes	Type I error rate of 0.05 (two-sided); no adjustments for biases at interim analysis	Yes, mentioned in title, methods, and results	All analyses performed using R software
Mastroianni et al. (2022) [20]	Covariate-adaptive randomization	None specified	NR	None	No	NR	Not reported	Yes, 80% power to detect 5% difference in primary outcome; based on previous studies	1:1 based upon BMI, ASA score, baseline hemoglobin, planned urinary diversion, neoadjuvant chemotherapy, and clinical tumour stage	1. Outcome assessors (i.e., pathologists)	Details not reported	Type I error rate of 0.05; no further details reported	Yes, mentioned in methods	All analysis performed using SPSS
Metcalfe et al. (2022) [21]	Group sequential; sample size re-estimation; covariate-adaptive randomization	1. Sample size re-estimation 2. Two pre-specified blinded interim analyses: 3. First interim analysis done for futility 4. Second interim analysis done for futility and efficacy	Sample size re-estimation	Recruitment and randomisation stopped after futility boundary was crossed at the first interim analysis	Yes, performed at start of trial	To determine predefined interim stopping threshold	Not reported	Yes, 90% power to detect a 6-point MCID with 15% loss to follow-up rate; based on previous studies	1:1 based upon site, sex, age, and cuff tear size	1. Patients (blinded for 12 months) and outcome assessors	1. mITT for primary outcome (adjusted for interim analysis) 2. Low rate of missing data so did not impute missing data points	Type I error rate of 0.05 (two-sided); adjustment for bias planned if study progressed beyond first interim analysis	Yes, mentioned in title, methods, and results	All analyses performed using R software
Neuberger et al. (2023) [22]	Group sequential; sample size re-estimation	1. Sample size re-estimation 2. One pre-specified interim analysis for futility and efficacy	1. Trial termination for futility or efficacy 2. Sample size re-estimation	Primary interim analysis: Sample size was increased	No	NR	NR	Yes, based on previous studies	1:1	1. Surgeons unblinded after surgery 2. Patients and assessors blinded until study completion	Yes	Type I error rate of 0.025 (one sided); no adjustments for biases at interim analysis	Yes, mentioned in methods and results	All analyses performed using JMP and R software
Reardon et al. (2017) [23]	Bayesian design and analysis	1. Bayesian analytical methods for non-inferiority 2. Bayesian interim analysis when 1400 patients reached 12-month follow-up	Sample size re-estimation	Primary interim analysis: No stated methodological changes	Yes, performed at start of trial	To determine Bayesian posterior probability for non-inferiority	Yes	Yes, performed using Bayesian analysis and based upon 17% incidence rate of primary outcome	1:1 stratified by site and need for revascularization	Open label	1. mITT for primary and secondary outcomes 2. Bayesian analogues of frequentist tests and posterior probabilities	Type I error rate of 0.05 (two-sided) used to calculate relevant Bayesian posterior probability for hypothesis testing	Yes, mentioned in methods and results	All analyses performed in conjunction with independent statistical group
Yoshioka et al. (2018) [24]	Adaptive random assignment procedure	NR	NR	NR	NR	NR	NR	NR	NR	NR	NR	No	Yes, mentioned in methods	Not specified

ITT, intention-to-treat; mITT, modified intention-to-treat; NA, not applicable; NR, not reported

### Risk of bias and CONSORT reporting

**Fig 2:**
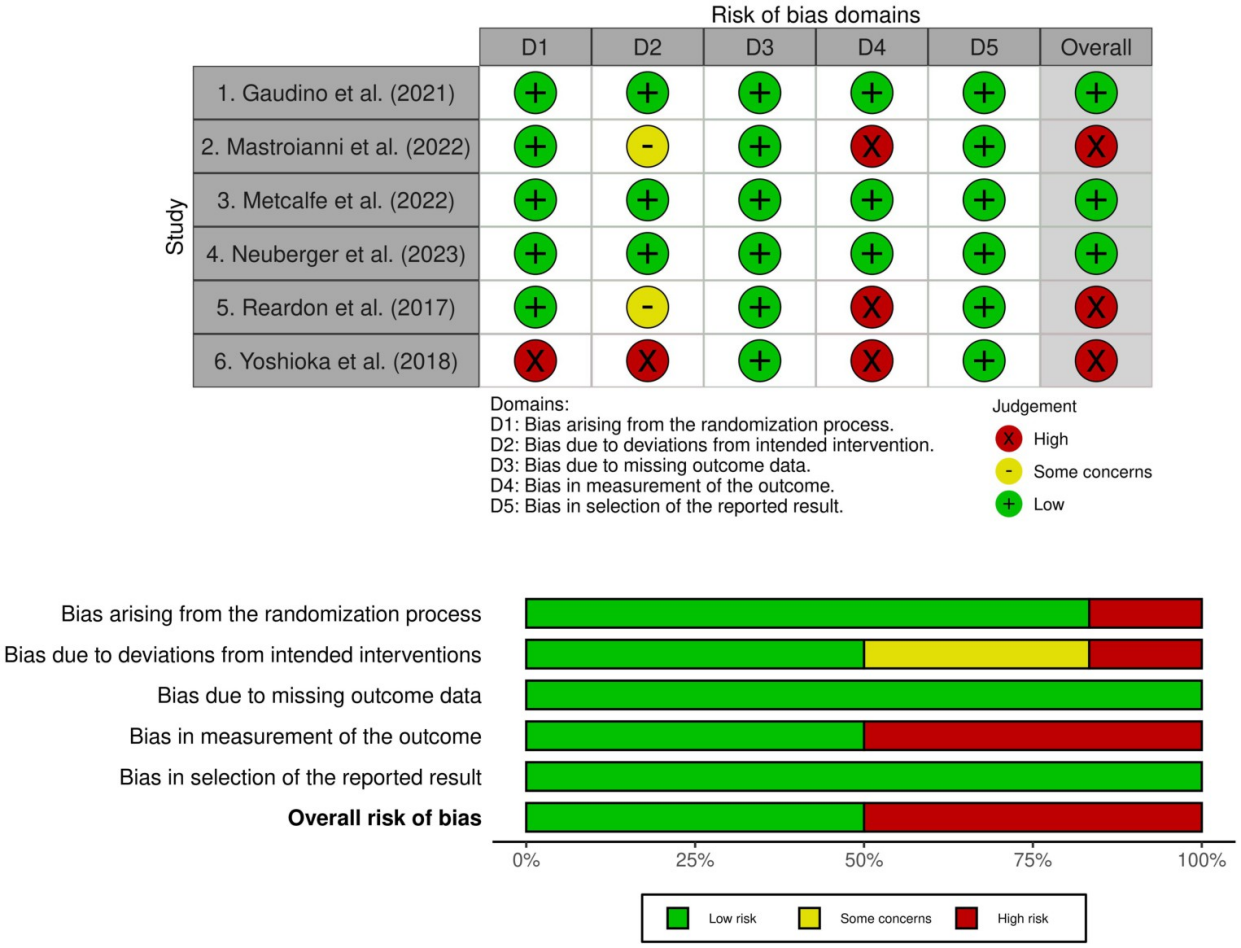
Risk of bias assessment for included studies.

**Fig 3:**
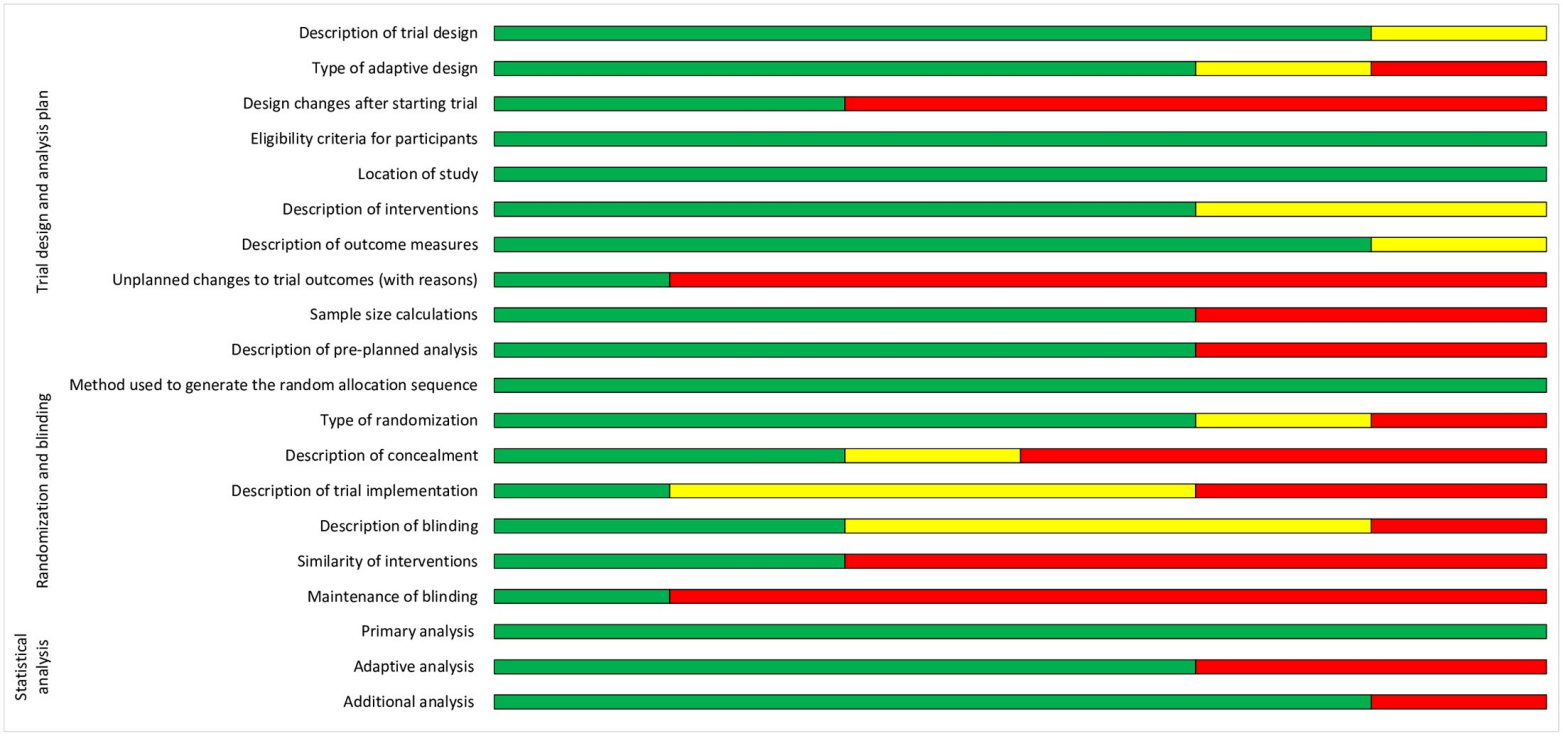
CONSORT ACE 2020 assessment of methodology reporting for included studies. Green = Fully reported; Yellow = Partially reported; Red = Not reported.

All trials were evaluated by two independent reviewers in duplicate using the Cochrane RoB 2.0 instrument (Fig 2). Overall, three (50%) trials had a low risk of bias and three (50%) had a high risk of bias. A high risk of bias was primarily derived from the randomization process, deviations from intended interventions, and the reporting of outcomes [20, 23, 24]. We also performed an assessment of reporting quality using the CONSORT extension ACE checklist [17]. Two studies directly referenced CONSORT ACE 2020 guidelines [20, 22]. Most studies (83.3%) reported trial registration, protocol, full statistical analysis plan, and funding sources. We described abstract and main text reporting outcomes for all included studies (S1 Table; S3 File). Despite other studies adequately reporting details for rationale, methods, and results, one study reported adaptive design details within their abstract [21]. We found that within the methods section of the main text, most reporting heterogeneity (i.e., 50–83.3% of studies describing either partial or no reporting) occurred when describing design changes following trial initiation, changes to study design after the start of the trial, and details regarding blinding implementation and adherence (Fig 3). In the results section, no studies reported the reasons for trial stoppage or sufficient details surrounding this decision, and there was heterogeneity in the quality of outcome reporting. Lastly, regarding the discussion, we found that findings were adequately contextualized, but only two studies fully reported study limitations with direct reference to adaptive design decisions [21, 22].

## Discussion

We present the first scoping review of adaptive clinical trials in surgery. Since 2015, six adaptive trials have been published comparing at least one surgical intervention, which only represents 0.5% of the database search. We also note that three trials recruited under 150 patients. Adaptive methodologies are increasingly being adopted in non-surgical trials, but their design remains nebulous and as such, they have seldom been applied to surgical populations [13].

Adaptive randomization methods represent an early adaptive method applied to trial design in medicine [25, 26]. Response-adaptive randomization can minimize the number of patients randomized to an inferior treatment arm and may suit multi-arm trials or trials with prolonged recruitment and moderately delayed responses,[27, 28]. This method of randomization may improve surgical trial recruitment and ethicality, as patients will have an increased probability of being randomized to an effective surgical arm [28]. Sirkis et al. (2022) simulated adaptive randomization techniques in the RECOVERY trial demonstrating that adaptive approaches may have reduced death and increased the likelihood of randomization to effective COVID-19 therapies [29]. Adaptive randomization has the potential, however, to optimize patient recruitment in surgical trials, but must be used prudently, as surgical trials are often small-to-moderate in size and therefore prone to prognostic imbalances. *A priori* trial simulation studies to guide sample size calculations and evaluate optimal adaptive randomization methods and their biases may further inform adaptive designs for surgical trials.

Outside of adaptive randomization alone, we found that three studies employed a group sequential design with blinded sample size re-estimation performed at one or two prespecified interim analyses [19, 21, 22]. A recent systematic review identified 27 adaptive trials that utilized sample size re-estimation, in particular, this design was used in Phase I/II trials wherein there was insufficient prior knowledge of treatment group [30]. Sample size re-estimation, however, can lead to an inflated type I error rate, and though methods for optimizing these statistical adaptations exist, they remain poorly standardized within the adaptive trial literature [31]. Group sequential trial designs are defined by at least one interim analysis being built into the trial design to evaluate efficacy and/or futility of treatment arms and can facilitate the adding or dropping of additional treatment arms [32]. Sequential designs have been used in pharmaceutical trials for decades, and more recently were employed in the DEVELOP-UK trial evaluating lung perfusion following lung transplantation [32, 33]. Two studies in this review reported that statistical simulations were performed to guide design and interim analyses [21, 23]. Statistical simulations represent another advantage of adaptive trials, as they can estimate efficiency gains and facilitate trial improvements prior to funding expenditure and beginning patient recruitment [34]. Reardon et al. (2017) utilized a Bayesian analysis plan to design and perform analysis within their trial [23]. Though statistically more complicated, Bayesian-designed trials hold promise in comparative effectiveness trials wherein prior population knowledge is lacking, as these designs can assist sample size estimations, improve power, and potentially increase the rate at which patients are randomized to effective treatments [35]. Current barriers to adaptive trial adoption amongst stakeholders, however, include patient consent, risks for bias, type I error rate, lack of clear rationale, and a paucity of education about adaptive methodologies [36].

Interestingly, combining adaptive methods such as sample size re-estimation couched within a response-adaptive RCT, may also prove helpful as the synergized application of these methods can further improve power and shorten trial duration [37]. Reardon et al. (2017) reported that an independent statistical group assisted with their analysis [23]. Better adoption of complex adaptive trial methodologies amongst surgeons will need to occur alongside improved biostatistical training for surgeons and increased collaboration with statisticians [38, 39]. Moreover, it is not difficult to conceptualize the interaction that can exist between generative artificial intelligence and the computations that underly adaptive trial design and execution [40].

We performed a quality appraisal using the Cochrane Rob 2.0 instrument for RCTs [18]. We were unable to identify a quality appraisal tool tailored to adaptive trial methodologies, but our research group is currently in the process of creating a customized adaptive trial RoB instrument. Our quality appraisal identified 50% of the included trials to have a high risk of bias, which was primarily derived from randomization details and inadequate outcome reporting. We used the CONSORT ACE 2020 guidelines to assess reporting quality in all included trials [17]. Within the main text, heterogenous reporting was primarily identified in the description of trial design, changes following the start of the trial, and details regarding blinding and concealment maintenance. These deficiencies are important to highlight, as adherence to pre-specified trial design parameters and interim analyses are critical in minimizing type I error rate and bias within adaptive methodologies. It will be important for future adaptive trials to adhere to the published CONSORT ACE 2020 guidelines. Our study limitations are the exclusion of any abstracts or grey literature, and despite liberal search criteria, the risk of missing any relevant surgical trials.

Surgical subspecialties are beginning to explore the role for adaptive trials in their respective disciplines [41, 42]. As we peer into the future of evidence-based surgery, we must identify the existing barriers to adoption and global dissemination of surgical trial design and implementation. For instance, our group is currently exploring the importance of pilot trials in surgery to overcome issues of early trial termination [5, 43]. As researchers characterize the importance of creativity in surgical innovation, we posit that adaptive trial methodologies provide yet another methodological tool to answer translational questions and advance evidence-based surgical knowledge [44]. Since conventional RCTs are often conducted out of high-income countries, cost-effective and efficient adaptive methodologies may facilitate practice-changing trials being conducted in low-middle income countries, thereby improving global collaboration and conclusion generalizability [45].

Adaptive designs have the potential to optimize patient recruitment and statistical power when sample sizes are small, or little is known about the research populations under investigation: common issues encountered in surgical trials [46]. These designs may also assist with conducting trials in rare disease populations [47]. Like any technological advancement, however, it is imperative that we always identify the appropriate research question and clinical scenario where adaptive methodologies can be best deployed, as they are not without their shortcomings [48]. For instance, adaptive trials are likely not best suited when there is a notable delay in outcome assessment or there is inadequate trial infrastructure at the primary institution. An adaptive design is, however, likely best suited when the gain in trial efficiency and cost-effectiveness greatly outweighs the added complexity to trial methodology and statistical analysis [48]. Here, we demonstrate that the number of published adaptive surgical trials is low and reporting of complex adaptive methods is often heterogenous and inadequate. Future adaptive trials must be reported in accordance with published CONSORT ACE 2020 guidelines [17]. As these methodologies continue to be optimized, we suggest that surgical trialists consider implementing adaptive design components when deemed appropriate for their clinical question and population-of-interest. Adaptive trial designs may help to improve the quality of surgical evidence, streamline time to reporting, and compliment the accelerated pace of innovation in surgery.

## Supporting information

S1 File Ovid search algorithm. (DOCX)

S2 File Raw data for all included trials. (XLSX)

S3 File Raw data for CONSORT ACE 2020 evaluation. (XLSX)

S1 Table CONSORT ACE 2020 assessment for included studies. (DOCX)

S1 Checklist Preferred Reporting Items for Systematic reviews and Meta-Analyses extension for Scoping Reviews (PRISMA-ScR) checklist. (DOCX)

## References

[1] Cook JA. The challenges faced in the design, conduct and analysis of surgical randomised controlled trials. Trials. 2009;10(1):9. doi: 10.1186/1745-6215-10-9 19200379 PMC2654883

[2] Catala-Lopez F, Aleixandre-Benavent R, Caulley L, Hutton B, Tabares-Seisdedos R, Moher D, Alonso-Arroyo A. Global mapping of randomised trials related articles published in high-impact-factor medical journals: a cross-sectional analysis. Trials. 2020;21(1):34. Epub 20200107. doi: 10.1186/s13063-019-3944-9 . 31910857 PMC6947860

[3] Wente MN, Seiler CM, Uhl W, Buchler MW. Perspectives of evidence-based surgery. Dig Surg. 2003;20(4):263–9. Epub 20030515. doi: 10.1159/000071183 . 12748428

[4] Pronk AJM, Roelofs A, Flum DR, Bonjer HJ, Abu Hilal M, Dijkgraaf MGW, et al. Two decades of surgical randomized controlled trials: worldwide trends in volume and methodological quality. Br J Surg. 2023;110(10):1300–8. doi: 10.1093/bjs/znad160 . 37379487 PMC10480038

[5] Rosenthal R, Kasenda B, Dell-Kuster S, von Elm E, You J, Blumle A, et al. Completion and Publication Rates of Randomized Controlled Trials in Surgery: An Empirical Study. Ann Surg. 2015;262(1):68–73. doi: 10.1097/SLA.0000000000000810 . 24979608

[6] Robinson NB, Fremes S, Hameed I, Rahouma M, Weidenmann V, Demetres M, et al. Characteristics of Randomized Clinical Trials in Surgery From 2008 to 2020: A Systematic Review. JAMA Netw Open. 2021;4(6):e2114494. Epub 20210601. doi: 10.1001/jamanetworkopen.2021.14494 . 34190996 PMC8246313

[7] Cook JA. The challenges faced in the design, conduct and analysis of surgical randomised controlled trials. Trials. 2009;10:9. Epub 20090206. doi: 10.1186/1745-6215-10-9 . 19200379 PMC2654883

[8] Farrokhyar F, Karanicolas PJ, Thoma A, Simunovic M, Bhandari M, Devereaux PJ, et al. Randomized controlled trials of surgical interventions. Ann Surg. 2010;251(3):409–16. doi: 10.1097/SLA.0b013e3181cf863d . 20142732

[9] Miller FG. The enduring legacy of sham-controlled trials of internal mammary artery ligation. Prog Cardiovasc Dis. 2012;55(3):246–50. doi: 10.1016/j.pcad.2012.09.002 . 23217427

[10] Sydes MR, Parmar MK, Mason MD, Clarke NW, Amos C, Anderson J, et al. Flexible trial design in practice—stopping arms for lack-of-benefit and adding research arms mid-trial in STAMPEDE: a multi-arm multi-stage randomized controlled trial. Trials. 2012;13:168. Epub 20120915. doi: 10.1186/1745-6215-13-168 . 22978443 PMC3466132

[11] Pushpakom S, Kolamunnage-Dona R, Taylor C, Foster T, Spowart C, Garcia-Finana M, et al. TAILoR (TelmisArtan and InsuLin Resistance in Human Immunodeficiency Virus [HIV]): An Adaptive-design, Dose-ranging Phase IIb Randomized Trial of Telmisartan for the Reduction of Insulin Resistance in HIV-positive Individuals on Combination Antiretroviral Therapy. Clin Infect Dis. 2020;70(10):2062–72. doi: 10.1093/cid/ciz589 . 31425580 PMC7201422

[12] Gerety EL, Lawrence EM, Wason J, Yan H, Hilborne S, Buscombe J, et al. Prospective study evaluating the relative sensitivity of 18F-NaF PET/CT for detecting skeletal metastases from renal cell carcinoma in comparison to multidetector CT and 99mTc-MDP bone scintigraphy, using an adaptive trial design. Annals of Oncology. 2015;26(10):2113–8. doi: 10.1093/annonc/mdv289 . 26202597 PMC4576907

[13] Pallmann P, Bedding AW, Choodari-Oskooei B, Dimairo M, Flight L, Hampson LV, et al. Adaptive designs in clinical trials: why use them, and how to run and report them. BMC Med. 2018;16(1):29. Epub 20180228. doi: 10.1186/s12916-018-1017-7 . 29490655 PMC5830330

[14] Stallard N, Hampson L, Benda N, Brannath W, Burnett T, Friede T, et al. Efficient Adaptive Designs for Clinical Trials of Interventions for COVID-19. Stat Biopharm Res. 2020;12(4):483–97. Epub 20200729. doi: 10.1080/19466315.2020.1790415 . 34191981 PMC8011600

[15] McGowan J, Straus S, Moher D, Langlois EV, O’Brien KK, Horsley T, et al. Reporting scoping reviews-PRISMA ScR extension. J Clin Epidemiol. 2020;123:177–9. Epub 20200327. doi: 10.1016/j.jclinepi.2020.03.016 . 32229248

[16] Macdonald M, Martin Misener R, Weeks L, Helwig M. Covidence vs Excel for the title and abstract review stage of a systematic review. International Journal of Evidence-Based Healthcare. 2016;14(4):200–1. doi: 10.1097/01.XEB.0000511346.12446.f2

[17] Dimairo M, Pallmann P, Wason J, Todd S, Jaki T, Julious SA, et al. The adaptive designs CONSORT extension (ACE) statement: a checklist with explanation and elaboration guideline for reporting randomised trials that use an adaptive design. Trials. 2020;21(1):528. Epub 20200617. doi: 10.1186/s13063-020-04334-x . 32546273 PMC7298968

[18] Sterne JAC, Savovic J, Page MJ, Elbers RG, Blencowe NS, Boutron I, et al. RoB 2: a revised tool for assessing risk of bias in randomised trials. BMJ. 2019;366:l4898. Epub 20190828. doi: 10.1136/bmj.l4898 . 31462531

[19] Gaudino M, Sanna T, Ballman KV, Robinson NB, Hameed I, Audisio K, et al. Posterior left pericardiotomy for the prevention of atrial fibrillation after cardiac surgery: an adaptive, single-centre, single-blind, randomised, controlled trial. Lancet. 2021;398(10316):2075–83. doi: 10.1016/S0140-6736(21)02490-9 Corporate Author: PALACS Investigators. Language: English. Entry Date: 20220108. Revision Date: 20220211. Publication Type: Journal Article. 34788640

[20] Mastroianni R, Tuderti G, Anceschi U, Bove AM, Brassetti A, Ferriero M, et al. Open vs robot-assisted radical cystectomy with totally intracorporeal urinary diversion: perioperative outcomes from a single center randomised controlled trial. European Urology Open Science. 2021;32(Supplement 1):S143.

[21] Metcalfe A, Parsons H, Parsons N, Brown J, Gemperle Mannion E, Haque A, et al. Subacromial balloon spacer for irreparable rotator cuff tears of the shoulder (START:REACTS): a group-sequential, double-blind, multicentre randomised controlled trial. The Lancet. 2022;399(10339):1954–63. doi: 10.1016/S0140-6736(22)00652-3 35461618

[22] Neuberger M, Kowalweski KF, Simon V, Wessels F, Siegel F, Worst TS, et al. A randomised controlled adaptive design phase III trial comparing a peritoneal flap versus no flap for lymphocele prevention after robotic-assisted radical prostatectomy with pelvic lymph node dissection: The PELYCAN study. European Urology. 2023;83(Supplement 1):S929–S31.

[23] Reardon MJ, Van Mieghem NM, Popma JJ, Kleiman NS, Sondergaard L, Mumtaz M, et al. Surgical or Transcatheter Aortic-Valve Replacement in Intermediate-Risk Patients. N Engl J Med. 2017;376(14):1321–31. Epub 20170317. doi: 10.1056/NEJMoa1700456 . 28304219

[24] Yoshioka I, Khanal A, Tominaga K, Horie A, Furuta N, Fukuda J. Vertical ramus versus sagittal split osteotomies: comparison of stability after mandibular setback. Journal of Oral & Maxillofacial Surgery (02782391). 2008;66(6):1138–44. doi: 10.1016/j.joms.2007.09.008 Language: English. Entry Date: 20080627. Revision Date: 20200708. Publication Type: Journal Article. 18486778

[25] Lim CY, In J. Randomization in clinical studies. Korean J Anesthesiol. 2019;72(3):221–32. Epub 20190401. doi: 10.4097/kja.19049 . 30929415 PMC6547231

[26] Mahajan R, Gupta K. Adaptive design clinical trials: Methodology, challenges and prospect. Indian J Pharmacol. 2010;42(4):201–7. doi: 10.4103/0253-7613.68417 . 20927243 PMC2941608

[27] Proschan M, Evans S. Resist the Temptation of Response-Adaptive Randomization. Clin Infect Dis. 2020;71(11):3002–4. doi: 10.1093/cid/ciaa334 . 32222766 PMC7947972

[28] Lin J, Lin LA. A General Overview of Adaptive Randomization Design for Clinical Trials. Journal of Biometrics & Biostatistics. 2016;07(02). doi: 10.4172/2155-6180.1000294

[29] Sirkis T, Jones B, Bowden J. Should RECOVERY have used response adaptive randomisation? Evidence from a simulation study. BMC Med Res Methodol. 2022;22(1):216. Epub 20220806. doi: 10.1186/s12874-022-01691-w . 35933340 PMC9356442

[30] Mano H, Tanaka Y, Orihara S, Moriya J. Application of sample size re-estimation in clinical trials: A systematic review. Contemp Clin Trials Commun. 2023;36:101210. Epub 20230918. doi: 10.1016/j.conctc.2023.101210 . 37842317 PMC10568275

[31] Liu Y, Xu H. Sample size re-estimation for pivotal clinical trials. Contemp Clin Trials. 2021;102:106215. Epub 20201118. doi: 10.1016/j.cct.2020.106215 . 33217555

[32] Potvin D, DiLiberti CE, Hauck WW, Parr AF, Schuirmann DJ, Smith RA. Sequential design approaches for bioequivalence studies with crossover designs. Pharm Stat. 2008;7(4):245–62. doi: 10.1002/pst.294 . 17710740

[33] Fisher A, Andreasson A, Chrysos A, Lally J, Mamasoula C, Exley C, et al. An observational study of Donor Ex Vivo Lung Perfusion in UK lung transplantation: DEVELOP-UK. Health Technol Assess. 2016;20(85):1–276. doi: 10.3310/hta20850 . 27897967 PMC5136735

[34] Li W, Cornelius V, Finfer S, Venkatesh B, Billot L. Adaptive designs in critical care trials: a simulation study. BMC Medical Research Methodology. 2023;23(1). doi: 10.1186/s12874-023-02049-6 37853343 PMC10585789

[35] Connor JT, Elm JJ, Broglio KR, Esett, Investigators A-I. Bayesian adaptive trials offer advantages in comparative effectiveness trials: an example in status epilepticus. J Clin Epidemiol. 2013;66(8 Suppl):S130–7. doi: 10.1016/j.jclinepi.2013.02.015 . 23849147 PMC3743558

[36] Madani Kia T, Marshall JC, Murthy S. Stakeholder perspectives on adaptive clinical trials: a scoping review. Trials. 2020;21(1):539. Epub 20200617. doi: 10.1186/s13063-020-04466-0 . 32552852 PMC7301522

[37] Li X, Hu F. Sample size re‐estimation for response‐adaptive randomized clinical trials. Pharmaceutical Statistics. 2022;21(5):1058–73. doi: 10.1002/pst.2199 35191605

[38] Lakhlifi C, Lejeune FX, Rouault M, Khamassi M, Rohaut B. Illusion of knowledge in statistics among clinicians: evaluating the alignment between objective accuracy and subjective confidence, an online survey. Cogn Res Princ Implic. 2023;8(1):23. Epub 20230420. doi: 10.1186/s41235-023-00474-1 . 37081292 PMC10118231

[39] Williams PJ, Murphy P, Van Koughnett JAM, Ott MC, Dubois L, Allen L, et al. Statistical Techniques in General Surgery Literature: What Do We Need to Know? J Am Coll Surg. 2018;227(4):450–4 e1. Epub 20180730. doi: 10.1016/j.jamcollsurg.2018.07.656 . 30071305

[40] Askin S, Burkhalter D, Calado G, El Dakrouni S. Artificial Intelligence Applied to clinical trials: opportunities and challenges. Health Technol (Berl). 2023;13(2):203–13. Epub 20230228. doi: 10.1007/s12553-023-00738-2 . 36923325 PMC9974218

[41] Jansen JO, Pallmann P, Maclennan G, Campbell MK. Bayesian clinical trial designs: Another option for trauma trials? Journal of Trauma and Acute Care Surgery. 2017;83(4):736–41. doi: 10.1097/TA.0000000000001638 28930965

[42] Lang T. Adaptive Trial Design: Could We Use This Approach to Improve Clinical Trials in the Field of Global Health? The American Society of Tropical Medicine and Hygiene. 2011;85(6):967–70. doi: 10.4269/ajtmh.2011.11-0151 22144428 PMC3225172

[43] Fairhurst K, Blazeby JM, Potter S, Gamble C, Rowlands C, Avery KNL. Value of surgical pilot and feasibility study protocols. Br J Surg. 2019;106(8):968–78. Epub 20190510. doi: 10.1002/bjs.11167 . 31074503 PMC6618315

[44] Thabane A, Busse JW, Sonnadara R, Bhandari M. Investigating divergent thinking and creative ability in surgeons (IDEAS): a survey protocol. BMJ Open. 2023;13(4):e069873. Epub 20230411. doi: 10.1136/bmjopen-2022-069873 . 37041058 PMC10106042

[45] Drain PK, Parker RA, Robine M, Holmes KK, Bassett IV. Global migration of clinical research during the era of trial registration. PLoS One. 2018;13(2):e0192413. Epub 20180228. doi: 10.1371/journal.pone.0192413 . 29489839 PMC5830297

[46] Metcalfe A, Parsons H, Parsons N, Brown J, Fox J, Gemperle Mannion E, et al. Subacromial balloon spacer for irreparable rotator cuff tears of the shoulder (START:REACTS): a group-sequential, double-blind, multicentre randomised controlled trial. Lancet. 2022;399(10339):1954–63. doi: 10.1016/S0140-6736(22)00652-3 . 35461618

[47] May M. Rare-disease researchers pioneer a unique approach to clinical trials. Nat Med. 2023;29(8):1884–6. doi: 10.1038/s41591-023-02333-4 . 37147501

[48] Wason JMS, Brocklehurst P, Yap C. When to keep it simple—adaptive designs are not always useful. BMC Med. 2019;17(1):152. Epub 20190802. doi: 10.1186/s12916-019-1391-9 . 31370839 PMC6676635

