# Supplementary material for: Adaptive clinical trials in surgery: A scoping review of methodological and reporting quality
Source: PLoS One. 2024 May 28;19(5):e0299494. doi: 10.1371/journal.pone.0299494 (PMC11132449; doi:10.1371/journal.pone.0299494)
Supplement: S1 File — (DOCX) [file pone.0299494.s001.docx]

**S1 File. Ovid Search algorithm**

1 Adaptive Clinical Trials as Topic/ 92

2 Adaptive Clinical Trial/ 36

3 ((adaptive or bayesian) adj2 (design* or trial* or random*)).ti. 1461

4 (adaptive clinical trial* or adaptive design* or adaptive random* or bayesian design*).ab. 1724

5 Continual Reassessment Method*.ti,ab. 386

6 dose response model*.ti,ab. 1704

7 flexible design*.ti,ab. 595

8 frequentist adapt*.ti,ab. 3

9 seamless phase*.ti,ab. 99

10 biomarker adapt*.ti,ab. 16

11 multi arm multi stage.ti,ab. 84

12 sample size re-estim*.ti,ab. 140

13 1 or 2 or 3 or 4 or 5 or 6 or 7 or 8 or 9 or 10 or 11 or 12 5401

14 exp Surgical Procedures, Operative/ 3560250

15 exp Specialties, Surgical/ 220795

16 surg*.tw,kf. 2409554

17 su.fs. 2270800

18 14 or 15 or 16 or 17 5244060

19 13 and 18 334
