# Supplementary material for: Adaptive clinical trials in surgery: A scoping review of methodological and reporting quality
Source: PLoS One. 2024 May 28;19(5):e0299494. doi: 10.1371/journal.pone.0299494 (PMC11132449; doi:10.1371/journal.pone.0299494)
Supplement: S1 Table — (DOCX) [file pone.0299494.s004.docx]

| **Supplementary Table 1.** CONSORT ACE 2020 assessment for included studies | | | | | | | | |
| --- | --- | --- | --- | --- | --- | --- | --- | --- |
|  | | | **Study** | | | | | |
|  |  |  | **Gaudino et al. (2021)** | **Mastroianni et al. (2022)** | **Metcalfe et al. (2022)** | **Neuberger et al. (2023)** | **Reardon et al. (2017)** | **Yoshioka et al. (2018)** |
| **ABSTRACT** | **Section/Topic** | **Standard CONSORT checklist item (extension for adaptive design)** | **Not cited** | **CONSORT cited** | **Not cited** | **CONSORT cited** | **Not cited** | **Not cited** |
|  | **Title** | Identification of study as randomised | Fully reported | Fully reported | Fully reported | Fully reported | Not reported | Not reported |
|  | **Authors** | Contact details for the corresponding author | Fully reported | Fully reported | Fully reported | Fully reported | Fully reported | Fully reported |
|  | **Trial design** | Description of the trial design (for example, parallel, cluster, non-inferiority); include the word “adaptive” in the content or at least as a keyword | Fully reported | Fully reported | Fully reported | Fully reported | Fully reported | Fully reported |
|  | **Methods** | Description of trial methods | Fully reported | Fully reported | Fully reported | Fully reported | Fully reported | Fully reported |
|  | **Participants** | Eligibility criteria for participants and the settings where the data were collected | Fully reported | Partially reported (setting not specified) | Fully reported | Not reported | Not reported | Not reported |
|  | **Interventions** | Interventions intended for each group | Fully reported | Fully reported | Fully reported | Fully reported | Fully reported | Fully reported |
|  | **Objective** | Specific objective or hypothesis | Fully reported | Fully reported | Fully reported | Fully reported | Fully reported | Not reported |
|  | **Outcome** | Clearly defined primary outcome for this report | Fully reported | Fully reported | Fully reported | Fully reported | Fully reported | Partially reported (no mention of primary outcome) |
|  | **Randomisation** | How participants were allocated to interventions | Fully reported | Fully reported | Fully reported | Fully reported | Not reported | Not reported |
|  | **Blinding (masking)** | Whether or not participants, care givers, and those assessing the outcomes were blinded to group assignment | Fully reported | Not reported | Fully reported | Fully reported | Not reported | Not reported |
|  | **Results** | -- | Fully reported | Fully reported | Fully reported | Fully reported | Fully reported | Partially reported (no statistics included) |
|  | **Numbers randomized** | Number of participants randomised to each group | Fully reported | Fully reported | Fully reported | Fully reported | Fully reported | Fully reported |
|  | **Recruitment** | Trial status | Fully reported | Fully reported | Fully reported | Fully reported | Fully reported | Not reported |
|  | **Adaptation decisions made** | Specify what trial adaptation decisions were made in light of the pre-planned decision-making criteria and observed accrued data | Not reported | Not reported | Fully reported | Not reported | Not reported | Not reported |
|  | **Numbers analysed** | Number of participants analysed in each group | Fully reported | Not reported | Fully reported | Not reported | Not reported | Fully reported |
|  | **Outcome** | For the primary outcome, a result for each group and the estimated effect size and its precision | Fully reported | Fully reported | Fully reported | Fully reported | Fully reported | Partially reported (only qualitative) |
|  | **Harms** | Important adverse events or side effects | Fully reported | Not reported | Partially reported (adverse events not specified) | Partially reported (adverse events not specified) | Fully reported | Not reported |
|  | **Conclusions** | General interpretation of the results | Fully reported | Fully reported | Fully reported | Fully reported | Fully reported | Fully reported |
|  | **Trial registration** | Registration number and name of trial register | Fully reported | Fully reported | Fully reported | Not reported | Fully reported | Not reported |
|  | **Funding** | Source of funding | Fully reported | Fully reported | Fully reported | Not reported | Fully reported | Not reported |
| **MAIN REPORT** | **Title and abstract** | Identification as a randomised trial in the title | Fully reported | Fully reported | Fully reported | Fully reported | Fully reported | Not reported |
|  |  | Structured summary of trial design, methods, results, and conclusions | Fully reported | Fully reported | Fully reported | Fully reported | Partially reported | Partially reported |
|  | **Introduction** |  |  |  |  |  |  |  |
|  | **Background and objectives** | Scientific background and explanation of rationale | Fully reported | Fully reported | Fully reported | Fully reported | Fully reported | Fully reported |
|  |  | Specific objectives or hypotheses | Fully reported | Fully reported | Fully reported | Fully reported | Fully reported | Fully reported |
|  | **Methods** |  |  |  |  |  |  |  |
|  | **Trial design** | Description of trial design (such as parallel, factorial) including allocation ratio | Fully reported | Fully reported | Fully reported | Fully reported | Fully reported | Partially reported (adaptive component not specified) |
|  |  | Type of adaptive design used, with details of the pre-planned trial adaptations and the statistical information informing the adaptations | Fully reported | Partially reported (details adaptability) | Fully reported | Fully reported | Fully reported | Not reported |
|  |  | Important changes to the design or methods after trial commencement (such as eligibility criteria) outside the scope of the pre-planned adaptive design features, with reasons | Fully reported | Not reported | Fully reported | Not reported | Not reported | Not reported |
|  | **Participants** | Eligibility criteria for participants | Fully reported | Fully reported | Fully reported | Fully reported | Fully reported | Fully reported |
|  |  | Settings and locations where the data were collected | Fully reported | Fully reported | Fully reported | Fully reported | Fully reported | Fully reported |
|  | **Interventions** | The interventions for each group with sufficient details to allow replication, including how and when they were administered | Fully reported | Partially reported (insufficient information for replication) | Fully reported | Fully reported | Fully reported | Partially reported (procedure steps incomplete) |
|  | **Outcomes** | Completely define pre-specified primary and secondary outcome measures, including how and when they were assessed. Any other outcome measures used to inform pre-planned adaptations should be described with the rationale | Fully reported | Fully reported | Fully reported | Fully reported | Fully reported | Partially reported (no pre-planned adaptation mentioned) |
|  |  | Any unplanned changes to trial outcomes after the trial commenced, with reasons | Not reported | Not reported | Fully reported | Not reported | Not reported | Not reported |
|  | **Sample size and operating characteristics** | How sample size and operating characteristics were determined | Fully reported | Not reported | Fully reported | Fully reported | Fully reported | Not reported |
|  |  | Pre-planned interim decision-making criteria to guide the trial adaptation process; whether decision-making criteria were binding or non-binding; pre-planned and actual timing and frequency of interim data looks to inform trial adaptations | Fully reported (no futility analysis done) | Not reported | Fully reported | Fully reported | Fully reported | Not reported |
|  | **Randomisation** |  |  |  |  |  |  |  |
|  | **Sequence generation** | Method used to generate the random allocation sequence | Fully reported | Fully reported | Fully reported | Fully reported | Fully reported | Fully reported |
|  |  | Type of randomisation; details of any restriction (such as blocking and block size); any changes to the allocation rule after trial adaptation decisions; any pre-planned allocation rule or algorithm to update randomisation with timing and frequency of updates | Fully reported | Fully reported | Fully reported | Fully reported | Partially reported (block size not mentioned) | Not reported |
|  | **Allocation concealment mechanism** | Mechanism used to implement the random allocation sequence (such as sequentially numbered containers), describing any steps taken to conceal the sequence until interventions were assigned | Fully reported | Not reported | Fully reported | Partially reported (concealment mechanism not specified) | Not reported | Not reported |
|  | **Implementation** | Who generated the random allocation sequence, who enrolled participants, and who assigned participants to interventions | Partially reported (team responsibilities not specified) | Partially reported (team responsibilities not specified) | Fully reported | Partially reported (team responsibilities not specified) | Not reported | Not reported |
|  | **Blinding** | If done, who was blinded after assignment to interventions (for example, participants, care providers, those assessing outcomes) and how | Fully reported | Partially reported (method not specified) | Fully reported | Partially reported (method not specified) | Partially reported (method not specified) | Not reported |
|  |  | If relevant, description of the similarity of interventions | Fully reported | Not reported | Fully reported | Not reported | Not reported | Not reported |
|  |  | Measures to safeguard the confidentiality of interim information and minimise potential operational bias during the trial | Not reported | Not reported | Fully reported | Not reported | Not reported | Not reported |
|  | **Statistical methods** | Statistical methods used to compare groups for primary and secondary outcomes, and any other outcomes used to make pre-planned adaptations | Fully reported | Fully reported | Fully reported | Fully reported | Fully reported | Fully reported |
|  |  | For the implemented adaptive design features, statistical methods used to estimate treatment effects for key endpoints and to make inferences | Fully reported | Not reported | Fully reported | Fully reported | Fully reported | Not reported |
|  |  | Methods for additional analyses, such as subgroup analyses and adjusted analyses | Fully reported | Fully reported | Fully reported | Fully reported | Fully reported | Not reported |
|  | **Results** |  |  |  |  |  |  |  |
|  | **Participant flow** | For each group, the numbers of participants who were randomly assigned, received intended treatment, and were analysed for the primary outcome and any other outcomes used to inform pre-planned adaptations, if applicable | Fully reported | Fully reported | Fully reported | Fully reported | Fully reported | Fully reported |
|  |  | For each group, losses, and exclusions after randomisation, together with reasons | Fully reported | Fully reported | Fully reported | Fully reported | Fully reported | Fully reported |
|  | **Recruitment and adaptations** | Dates defining the periods of recruitment and follow-up, for each group | Fully reported | Fully reported | Fully reported | Fully reported | Fully reported | Fully reported |
|  |  | Why the trial ended or was stopped | Not reported | Not reported | Not reported | Not reported | Not reported | Not reported |
|  |  | Specify what trial adaptation decisions were made considering the pre-planned decision-making criteria and observed accrued data | Fully reported | Not reported | Fully reported | Fully reported | Partially reported  (method not specified) | Not reported |
|  | **Baseline data** | A table showing baseline demographic and clinical characteristics for each group | Fully reported | Fully reported | Fully reported | Fully reported | Fully reported | Fully reported |
|  |  | Summary of data to enable the assessment of similarity in the trial population between interim stages | Fully reported | Fully reported | Fully reported | Fully reported | Fully reported | Not reported (no interim stages) |
|  | **Numbers analysed** | For each group, number of participants (denominator) included in each analysis and whether the analysis was by original assigned groups. The number of participants by treatment group should be reported for each analysis at both the interim analyses and final analysis whenever a comparative assessment is performed | Fully reported | Fully reported | Fully reported | Fully reported | Fully reported | Fully reported |
|  | **Outcomes and estimation** | For each primary and secondary outcome, results for each group, and the estimated effect size and its precision (such as 95% confidence interval) | Fully reported | Fully reported | Fully reported | Fully reported | Fully reported | Partially reported (precision not addressed) |
|  |  | For binary outcomes, presentation of both absolute and relative effect sizes is recommended | Fully reported | Partially reported (only p values) | Fully reported | Fully reported | Fully reported | Partially reported (only p value) |
|  |  | Report interim results used to inform interim decision-making | Fully reported | Not reported (interim analysis done, but no effect on decision-making) | Fully reported | Partially reported (do not state the numbers explicitly) | Fully reported | Not reported |
|  | **Ancillary analyses** | Results of any other analyses performed, including subgroup analyses and adjusted analyses, distinguishing pre-specified from exploratory | Fully reported | Fully reported | Fully reported | Fully reported | Fully reported | Fully reported |
|  | **Harms** | All important harms or unintended effects in each group | Fully reported | Fully reported | Fully reported | Fully reported | Fully reported | Not reported |
|  | **Discussion** |  |  |  |  |  |  |  |
|  | **Limitations** | Trial limitations, addressing sources of potential bias, imprecision, and, if relevant, multiplicity of analyses. | Partially reported (design not addressed) | Partially reported (design not addressed) | Fully reported | Fully reported | Partially reported (design not addressed) | Not reported |
|  | **Generalisability** | Generalisability (external validity, applicability) of the trial findings. | Partially reported (briefly mention external validity) | Partially reported (briefly mention external validity) | Fully reported | Fully reported | Partially reported (do not mention external validity at all) | Not reported |
|  | **Interpretation** | Interpretation consistent with results, balancing benefits, and harms, and considering other relevant evidence | Fully reported | Fully reported | Fully reported | Fully reported | Fully reported | Partially reported (other relevant evidence not addressed) |
|  | **Other information** |  |  |  |  |  |  |  |
|  | **Registration** | Registration number and name of trial registry | Fully reported | Fully reported | Fully reported | Fully reported | Fully reported | Not reported |
|  | **Protocol** | Where the Full trial protocol can be accessed | Fully reported | Fully reported | Fully reported | Fully reported | Fully reported | Not reported |
|  | **SAP and other relevant trial documents** | Where the Full statistical analysis plan and other relevant trial documents can be accessed | Fully reported | Fully reported | Fully reported | Fully reported | Fully reported | Not reported |
|  | **Funding** | Sources of funding and other support (such as supply of drugs), role of funders | Fully reported | Fully reported | Fully reported | Fully reported | Fully reported | Not reported |
