# Supplementary material for: Adaptive clinical trials in surgery: A scoping review of methodological and reporting quality
Source: PLoS One. 2024 May 28;19(5):e0299494. doi: 10.1371/journal.pone.0299494 (PMC11132449; doi:10.1371/journal.pone.0299494)
Supplement: Attachment — Submitted filename: 3. PLOS One.docx [file pone.0299494.s006.docx]

PLOS One

**Adaptive clinical trials in surgery: A scoping review of methodological and reporting quality.**

**Summary:** There are no issues with the approach to the topic; I like the adaptive clinical trials and their review, as well as the approach used by the authors for the summary. The manuscript scores well for the novelty, significance, interest, and impact of the finding on the current state of RCTs in the literature. The authors have done extensive hard work on literature search and summarizing the information; very good descriptive analysis, well-organized results, and nicely covered discussion. The tables are compact, the flow chart is clear, and the methods are appropriately used. Overall, it has a good methodology and approach.

**Major Strengths:** This topic is very interesting and adds good knowledge/information to the upcoming researchers on how to address some concerns while conducting RCTs.
**Major Weaknesses:** NIL.

**Background/Introduction**

- Randomized controlled trials (RCTs) are the gold standard for evidence-based healthcare.
- **Problem**
  - Surgical trials suffer from high discontinuation and nonpublication rates, often due to slow patient recruitment.
  - several methodological concerns with surgical RCTs: small sample sizes, minor clinical outcomes, moderate-to-high methodological bias, and inconsistent usage of blinding and expertise-based randomization.
  - high costs, feasibility issues, between-group crossover, and poor patient adherence
- **Solution**

Adaptive trials can streamline study design and time-to-trial reporting.

- **Advantages**
  - flexible adjustments to the study protocol based upon pre-specified interim analyses, which can permit sample size re-calculations, adding or dropping treatment arms and/or stopping the trial for lack of efficacy.
  - Cost reduction, decreased probability of assigning patients to an ineffective treatment arm, and expedited trial completion
- **Objective:** a scoping review of the methodology and quality of adaptive surgical trials.

**MATERIALS AND METHODS**

**Study design**

- **Inclusion Criteria:** All Surgical adaptive RCTs (any Surgical pathway).
- Registration: Open Science Framework (DOI:10.17605/OSF.IO/2GDC5)
- The authors have followed PRISMA Scoping Review (PRISMA-ScR) guidelines.

**Search Strategy**

- librarian specialist
- Search engines: Medline (Ovid), EMBASE (Ovid), Web of Science, and Cochrane Collaboration from database inception to October 14th, 2023 (Appendix A).
- clinicaltrials.gov and pre-publication databases covered.
- Covidence used.

**Study Selection**

- Inclusion and Exclusion criteria defined.
- No bias in screening and data collection: Full-text review and data extraction, done by two people, with a third person for clarification.

**Outcomes of interest and data extraction**

- assess the methodological and reporting quality.
- Data collection: study design and adaptive trial methodologies.

**Quality appraisal**

- Two authors did this independently.
- Risk of bias assessment: Cochrane RoB 2.0
- Evaluation of reporting quality: CONSORT guidelines

**Statistical analysis**

- Statistical process: descriptive methods

**Results**

- Total studies identified: 1338 studies.
- six trials met the inclusion criteria. All were published after 2015.
- cardiothoracic and orthopedic surgery
- all of them prospective adaptive RCTs and one non-inferiority trial
- group sequential design with pre-specified interim analyses planned for efficacy, futility, and/or sample size re-estimation.

**Risk of bias and CONSORT reporting quality assessment**

- high risk of bias in 50% of included trials
- Reporting quality was heterogeneous: unplanned design changes, outcome assessments, randomization, and blinding concealment details.

**Conclusions**

- The authors suggest surgical trialists should consider implementing adaptive components to help improve patient recruitment and reduce trial duration.
